# Usability of a Culturally Informed mHealth Intervention for Symptoms of Anxiety and Depression: Feedback From Young Sexual Minority Men

**DOI:** 10.2196/humanfactors.7392

**Published:** 2017-08-25

**Authors:** John B Fleming, Yvette N Hill, Michelle Nicole Burns

**Affiliations:** ^1^ Center for Behavioral Intervention Technologies Department of Preventive Medicine Northwestern University Feinberg School of Medicine Chicago, IL United States; ^2^ IMPACT LGBT Health and Development Program Department of Medical Social Sciences Northwestern University Feinberg School of Medicine Chicago, IL United States

**Keywords:** mHealth, eHealth, homosexuality, male, adolescents, male, anxiety, depression

## Abstract

**Background:**

To date, we are aware of no interventions for anxiety and depression developed as mobile phone apps and tailored to young sexual minority men, a group especially at risk of anxiety and depression. We developed TODAY!, a culturally informed mobile phone intervention for young men who are attracted to men and who have clinically significant symptoms of anxiety or depression. The core of the intervention consists of daily psychoeducation informed by transdiagnostic cognitive behavioral therapy (CBT) and a set of tools to facilitate putting these concepts into action, with regular mood ratings that result in tailored feedback (eg, tips for current distress and visualizations of mood by context).

**Objective:**

The aim of this study was to conduct usability testing to understand how young sexual minority men interact with the app, to inform later stages of intervention development.

**Methods:**

Participants (n=9) were young sexual minority men aged 18-20 years (Mean=19.00, standard deviation [SD]=0.71; 44% black, 44% white, and 11.1% Latino), who endorsed at least mild depression and anxiety symptoms. Participants were recruited via flyers, emails to college lesbian, gay, bisexual, and transgender (LGBT) organizations, Web-based advertisements, another researcher’s database of sexual minority youth interested in research participation, and word of mouth. During recorded interviews, participants were asked to think out loud while interacting with the TODAY! app on a mobile phone or with paper prototypes. Feedback identified from these recordings and from associated field notes were subjected to thematic analysis using a general inductive approach. To aid interpretation of results, methods and results are reported according to the consolidated criteria for reporting qualitative research (COREQ).

**Results:**

Thematic analysis of usability feedback revealed a theme of general positive feedback, as well as six recurring themes that informed continued development: (1) functionality (eg, highlight new material when available), (2) personalization (eg, more tailored feedback), (3) presentation (eg, keep content brief), (4) aesthetics (eg, use brighter colors), (5) LGBT or youth content (eg, add content about coming out), and (6) barriers to use (eg, perceiving psychoeducation as homework).

**Conclusions:**

Feedback from usability testing was vital to understanding what young sexual minority men desire from a mobile phone intervention for symptoms of anxiety and depression and was used to inform the ongoing development of such an intervention.

## Introduction

### Mental Health and Sexual Minority Youth

Individuals identifying as lesbian, gay, or bisexual experience mental health disparities relative to the general population [1]. Gay men, specifically, experience mood and anxiety disorders at 2-3 times the rate of heterosexual men [2]. Similarly, young sexual minority men are at greater risk for anxiety and depressive symptoms than their heterosexual peers [3], and male sexual minority youth experience more associated symptoms than adult sexual minority men [4]. Additionally, anxiety and depression have been linked with human immunodeficiency virus (HIV) risk behavior (eg, condomless anal sex) among young sexual minority men (eg, [5-7]); this is especially salient given that rates of new HIV infections are particularly high in young sexual minority men aged 13-24 years, accounting for 24% of new HIV diagnoses among all sexual minority men and 91% of new HIV diagnoses in all men their age [8].

Adolescence and young adulthood may be a particularly challenging phase of development for sexual minority people. In the normal developmental process of identity formation and integration [9], these individuals also come to understand that their sexual orientation places them in a stigmatized minority. Developing an identity to incorporate that status, attempting to extricate one’s self concept from the societal stigma attached to it, and integrating this new identity into the whole self is a unique source of stress in this population [10]. Unfortunately, the extent to which existing psychotherapy protocols for anxiety or depression are efficacious among these young men is unknown. There is evidence, however, that psychological treatments culturally tailored for use with sexual minority men can produce results more quickly than standard cognitive behavioral therapy (CBT) (eg, by producing more rapid decreases in methamphetamine use among methamphetamine-dependent gay and bisexual men) [11]. There has been only limited research devoted to the development of psychological interventions for anxiety or depression that are specifically tailored for sexual minority youth and the factors that may drive their higher rates of distress.

### Interventions for This Population

To date, we are aware of only two such interventions in the research literature. One, a face-to-face intervention for young gay and bisexual men, was developed using an approach and guiding principles [12,13] similar to those we describe here. A small randomized controlled trial demonstrated some promising outcomes compared with a wait-list control [14]. However, the intervention was delivered to sexual minority men between the ages of 18-35 years, and thus, was not specific to youth. The other intervention, Rainbow SPARX, is a computer-based intervention for depression that conveys concepts from CBT to adolescents in the form of a game [15]. This intervention was adapted for sexual minority youth from an existing intervention designed for a general adolescent population (SPARX) [16]. An open pilot study demonstrated promising outcomes and indicated that Rainbow SPARX is acceptable to and feasible with sexual minority youth [17].

Technology may be a relatively inexpensive way to disseminate a culturally tailored evidence-based intervention to sexual minority men. Despite acknowledgment that population-specific clinical competencies are vital to providing care to these individuals, there is a shortage of psychologists adequately trained in these competencies [18-20]. Other barriers to care for this community include cost, privacy concerns, and stigma concerning mental health issues and sexual orientation [21]. An anxiety or depression intervention provided on a mobile phone platform would be privately and inexpensively accessible wherever and whenever its user might feel distressed and could help compensate for the lack of culturally competent clinicians. Among youth in particular, mobile technology may be especially promising given the pervasive use of mobile phones among this age group [22]. However, to our knowledge, there have been no studies examining the use of a mobile intervention for symptoms of anxiety and depression designed for young sexual minority men.

### The TODAY! App

To address this need, we developed TODAY!, a mobile phone app that offers young sexual minority men concrete steps they can use to more effectively manage anxiety and depressive symptoms. The frequent comorbidity of these symptoms and the cooccurring psychosocial problems sexual minority men frequently experience suggested that a transdiagnostic approach might be the most appropriate [12,23]. We therefore used concepts from transdiagnostic CBT [23] to inform the creation of the didactic modules and tools that comprise the core of TODAY! Many tools were designed to be presented to the user immediately after he reports a negative mood. The inclusion and development of these tools were heavily influenced by the concept of just-in-time interventions [24] and maximizing our ability to deliver tailored assistance to an individual at the moment he is experiencing distress.

Tailoring CBT concepts to this population required a working theory that accounted for the disparities in psychological distress experienced by these youth. These disparities are best understood in light of the minority stress theory that is well supported by the research literature. This theory posits that stress resulting from the extra burden of societal stigma is responsible for disparate rates of mental health concerns among sexual minority individuals [25,26]. This societal stigma includes discrimination, bullying, physical violence, and anti-gay public rhetoric, as well as the accumulation of microaggressions [27]. This stigma, when internalized by sexual minorities against themselves, is known as internalized homonegativity and becomes a significant stressor in its own right [28,29].

We took great care to be inclusive of the young sexual minority men for whom the intervention is intended. A recently published report that provides recommendations for tailoring eHealth interventions for sexual minority individuals supports our approach [30]. The recommendations highlight the need for examples, characters, and imagery, especially around relationships, that reflect the unique aspects of the intended audience’s lived experiences. TODAY! was designed with these young men’s unique experience of minority stress in mind by, for instance, utilizing examples to show how common CBT techniques (eg, problem solving or cognitive restructuring) can be applied to these stressors [31]. We did so without using labels or focusing on sexual identity, an approach the report also recommends [30] and which was one of the fundamental principles that informed the development of TODAY!. Finally, the report highlights the usefulness of including helplines and other resources for sexual minority people, both of which have been included in TODAY! since the earliest versions.

### This Study

To support the development of TODAY!, we conducted a series of usability testing sessions with young sexual minority men experiencing at least mild symptoms of anxiety or depression. The process of usability testing helped elucidate what this population desires from a mobile phone app designed to help young men like them cope with symptoms of anxiety and depression. During this study, we identified several recurring themes that shaped the design of the intervention as it underwent development.

## Methods

### Interpretation

To maximize transparency, we follow the consolidated criteria for reporting qualitative research (COREQ) in reporting our methods and results [32]. COREQ is a checklist of criteria, intended to be comprehensive, by which qualitative studies can be assessed and compared with one another and with which the results of qualitative studies can be better interpreted and understood in context [32]. The 32 items on the COREQ fall into three domains: (1) characteristics of the research team and their relationships with participants, (2) research study design, and (3) analysis and interpretation of data [32]. Addressing all COREQ criteria in our methods and results should aid interpretation by disclosing relevant details about the context in which the usability testing took place.

### Recruitment

Young sexual minority men were recruited in a large Midwestern city through (1) flyers placed in general neighborhood locations, as well as at community organizations frequented by sexual minority people; (2) Facebook advertisements targeting males who reported interest in other males; (3) Web-based advertisements in general venues, as well as venues dedicated to sexual minority people; and (4) another researcher’s database of sexual minority youth interested in research participation. Some youth also stated that they learned of the study through word of mouth. Interested youth completed a telephone screening with study staff to establish eligibility. Eligible participants were young cisgender men (ie, men who were assigned a male sex at birth and who presently identify as male) aged 17-20 years who endorsed sexual attraction to other males, experienced at least mild depressive or anxious symptoms per a verbally administered Patient Health Questionnaire for Depression and Anxiety 4-item scale (PHQ-4) [33] score of 3 or greater, and were familiar with the use of a mobile phone. Potential participants were excluded if they reported a psychiatric history that suggested that the intervention, once developed, might be insufficient to meet the youth’s needs or otherwise inappropriate (eg, a reported diagnosis of psychosis, post-traumatic stress disorder, substance dependence, or prior psychiatric hospitalization). We recruited participants in waves of 3-5, integrating participant feedback into the intervention on a rolling basis after each wave. We planned to conclude the study when three consecutive interviews failed to produce major new critiques or actionable suggestions.

### Intervention

**Figure 1:**
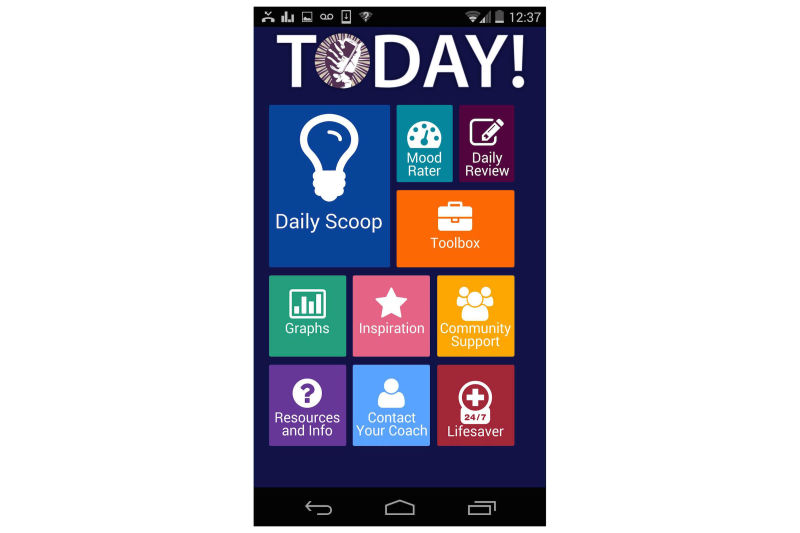
The TODAY! home screen.

**Figure 2:**
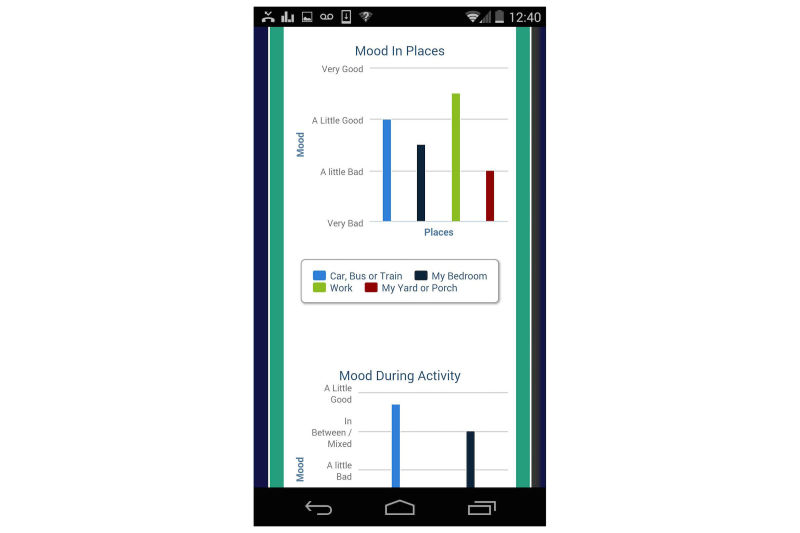
Mood variation by social context visualization.

**Figure 3:**
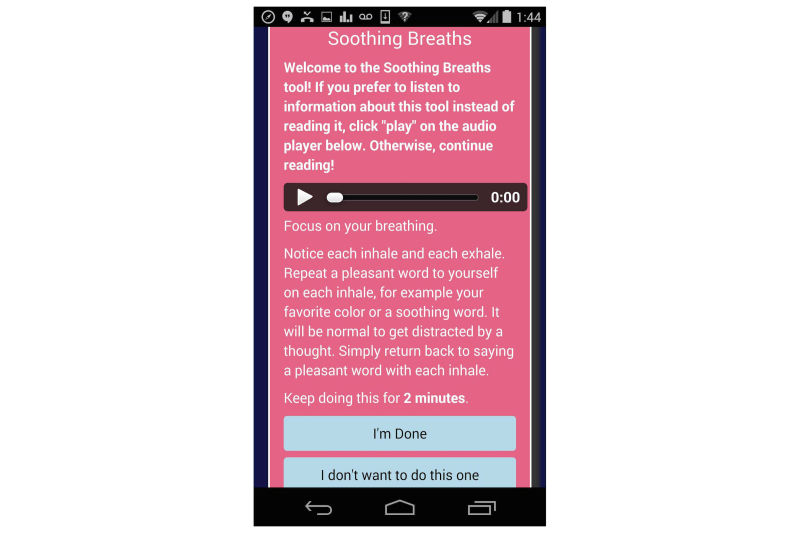
An In-the-Moment tool intended to help manage current anxiety.

TODAY! is a 10-week mobile phone-based intervention designed to target clinically significant symptoms of depression and anxiety. It consists of a mobile phone app (see Figure 1 for the home screen) and an accompanying coaching protocol. The app content is informed by transdiagnostic CBT protocols that focus on factors that are common across internalizing disorders such as emotion regulation and cognitive appraisals [23]. The app is culturally tailored for young sexual minority men and consists of (1) short, sequential daily didactics called Daily Scoops (50 in total) that familiarize users with cognitive behavioral concepts, tools, and skills; (2) the Toolbox, a set of interactive tools to facilitate putting those concepts into action (eg, a Thought Record); (3) prompts for mood ratings and social context several times per day; (4) a retrospective daily review of important events, including high points, low points, and coping strategies employed [34]; and (5) feedback. This feedback includes data visualizations intended to show the user how his reported mood varies by social context and time (see Figure 2). Feedback also includes In-the-Moment tools, or tips to help manage current distress. These In-the-Moment tools are accessible on demand in the Toolbox but are also offered to the user after a negative mood rating. For example, when a user rates their mood and indicates that they are angry, TODAY! presents the In-the-Moment tools designed to help manage anger. After the user indicates he has completed one of the In-the-Moment tools, mood is assessed again to see if there has been any improvement. Figure 3 shows one of the In-the-Moment tools.

Not all of the intervention content is available from the beginning. Daily Scoops and the tools in the Toolbox are hidden at the start and unlocked over time, sequentially. For the first 50 days, a new Daily Scoop is unlocked each day. New tools in the Toolbox are unlocked when the Daily Scoop that introduces them is unlocked. As the intervention is intended to be used over a 10-week period, no new material is presented during the last 20 days. This is intended to give users room to miss some material and still catch up during the intervention period.

Throughout the app, we employ examples pertinent to the target population, such as

coping with negative societal views on same-sex attraction or deciding if, when, and how to disclose their same-sex attraction to friends and family. The intervention is also supplemented with inspirational material intended to help combat internalized homonegativity, including inspirational quotes, accomplishments of influential sexual minority men, and affirming music videos. The intervention material is provided entirely within the TODAY! app but will be accompanied by weekly support (by telephone and email or text message [short message service, SMS]) from a master’s level clinician who will employ motivational interviewing [35] techniques with the aim of enhancing engagement with the app. As the coach is not available around the clock and some youth may find they need to talk to someone urgently in crisis situations such as suicidal ideation or dramatically losing family support, TODAY! includes a Lifesaver feature accessible from the main screen. The Lifesaver allows for quick access to telephone support 24 hours a day, 7 days a week through a national crisis hotline for sexual minority youth, a national suicide crisis hotline, and 911.

There may be some questions as to why the app itself was designed for a very specific subpopulation of sexual minority youth, that is, individuals assigned male sex at birth, who identify as male, and who are sexually attracted to other males. First, a primary purpose of developing this intervention is to provide a culturally tailored intervention. As the group being targeted by such an intervention is expanded, the amount of cultural targeting possible grows smaller. Second, evidence suggests that models of minority stress may differ between sexual minority subpopulations [36]. In light of this and in such a preliminary study of a novel intervention, we decided to reduce the heterogeneity of the sample by focusing on a specific subpopulation. If future studies suggest this intervention is effective, it would be reasonable to expect that similarly targeted interventions might be effective among other sexual minority populations.

### Usage Flow

Typical usage of the app would consist of consuming any newly available didactic material from the Daily Scoops at the start of the day, followed by practice use of any tools discussed or introduced in that material. Throughout the day, he would rate his mood periodically and use the suggested In-the-Moment tools as needed to improve mood. There is no intended frequency that the Toolbox should be accessed. However, to gain maximum benefit from the app, we encourage regular use. Toward the end of the day, a typical user would reflect on his day and complete the Daily Review. After using the app for several days, he might also start periodically checking the Graphs section to see the patterns the app has recorded regarding his mood and behavior. Usage of TODAY! features that are not part of the core intervention is likely to be somewhat idiosyncratic, with users accessing these features when they determine a personal need or interest.

### Procedure

Each youth who screened eligible for the study was invited to meet with us for one session, at their choice of our offices at an urban Midwestern university or at a local LGBT community center. All study procedures were approved by the university’s institutional review board (IRB). The sessions lasted approximately 3 hours, with an additional 30 min available for breaks if participants desired them. Upon the arrival of each participant, the researcher discussed the study with him and obtained informed consent. An assent procedure and waiver of parental consent were prepared and approved by the IRB for any participants who were 17 years of age. As no 17-year-old participants enrolled, we did not have occasion to use that procedure. To characterize the sample, participants completed the self-report Patient Health Questionnaire for Depression 9-item scale (PHQ-9) and the Generalized Anxiety Disorder 7-item scale (GAD-7) to measure depressive and anxious symptoms, respectively [37,38]. Each participant also filled out a brief demographic questionnaire and surveys of his typical Internet and mobile phone usage.

Next, participants completed a semistructured interview (see Multimedia Appendix 1) with research staff. First, the intervention components were verbally described to the participant to elicit the youth’s initial reaction to the concept and to discover which topics the participant believed would be helpful to include in such an intervention. Then, each participant interacted with various features of the app using paper prototypes or a partially functional version running on a mobile phone. This portion of the session was conducted using a think-aloud paradigm, where the youth were asked to verbalize the thoughts they were having, questions they had, and any decisions they were making (such as what to press on the screen when there were multiple actions that could be taken) as those thoughts, questions, or decisions occurred [39]. Afterwards, the semistructured interview resumed by asking each participant about his opinions after interacting with the app. To capture all the relevant data produced in a usability session, the interviews and think-aloud sessions were video recorded, with the exception of one session that was audio recorded due to technical issues. The interviewer kept field notes during the think-aloud exercise, and otherwise when appropriate, to supplement the recorded data. Sessions were conducted privately, with only the interviewer and participant present. Each participant received US $60 in cash, as well as travel cost reimbursement up to US $7 at the conclusion of his usability feedback session.

### Interviewers

The sessions were led by either a white, cisgender female Ph.D. level clinical psychologist and assistant professor (senior author) or a Hispanic, cisgender female predoctoral clinical psychology resident (second author). The senior author has previously conducted research with this population through partnerships with other researchers and institutes, and her perspective is informed by the minority stress theory [25,26]. Additionally, she has years of experience working with behavioral intervention technologies such as the one described here. The second author’s clinical psychology residency had a primary focus of research and clinical work with sexual and gender minority individuals. No participant was familiar with either interviewer before his usability session, nor did the format of the usability sessions allow for participants to learn about the interviewers’ personal motives for performing this research.

### Analysis

We chose to recruit participants in waves of 3-5, making any iterative changes to the intervention after each wave, such that at least three participants would review the same version of the intervention. We ended recruitment when three consecutive interviews produced no distinct major critiques or actionable suggestions. These were our chosen criteria for determining when data saturation had been reached. Descriptive statistics calculated on the self-report data provided details regarding the makeup of our sample. We then subjected the comments obtained during the usability feedback sessions to thematic analysis using a general inductive approach [40]. Having only one contact with each person, participants did not have an opportunity to review and possibly correct our raw data. We used no a priori codes or codebook; rather, we looked for themes that emerged from the data itself. First, the second author reviewed the recorded sessions and transcribed participant comments when they constituted an identifiable item of usability feedback. These transcribed comments were combined with observed participant behaviors and technical issues that the first author noted at the time of the interviews. The second author then used Microsoft Excel to sort this data into initial proposed thematic areas. All three authors then reviewed the data together to come to an initial consensus on what the major themes were. Finally, the first and senior author individually coded each item of feedback, assigning each to one of the identified themes. Items not coded identically by both coders were discussed until consensus was reached as to which category each piece of feedback fit into. The themes that emerged during this analysis informed continued development of the intervention. This means that later participants interacted with more refined versions of the app than previous participants did. Participants did not have an opportunity to respond to the findings of this study once analysis was complete.

## Results

### Participants

We reached data saturation after 9 young men participated in usability sessions. Of these 9 participants, 44.4% (4/9) identified as black, 44.4% (4/9) as white, and 11.1% (1/9) as Latino. Participant ages ranged from 18 to 20 (Mean=19.00, SD=0.71). All participants (N=9) reported an exclusively gay sexual identity. Eight youth reported being sexually attracted only to males, with one reporting being attracted mostly to males but to some females. All participants reported owning a mobile phone that was nearly always with them. The mean PHQ-9 score of 8.67 (SD 3.35) was over the 90th percentile according to German studies, with representative samples of male adolescents and young adults [41,42] Likewise, the mean score of 8.44 (SD=4.33) on the GAD-7 was in the 92nd percentile [43]. All participants who came to their scheduled session gave informed consent and participated fully.

### Usability Feedback

All participants expressed enthusiasm about an app created to help young sexual minority men like themselves with anxiety and depression. These general positive comments constituted one theme that emerged from our data. The analysis of more specific items of feedback revealed six more themes that, by highlighting what required improvement, informed the ongoing development of TODAY!: (1) Functionality, or comments concerning the features available in the app, as well as usability concerns such as navigation; (2) Personalization, or feedback regarding the extent to which interaction with the app was tailored to the individual and his circumstances based on input from the user; (3) Presentation, including the methods by which information was conveyed to the user; (4) Aesthetics, which covered the visual experience of using the app; (5) LGBT or Youth Content, comprised of suggestions of additional features or content that participants believed could benefit young sexual minority men; and (6) Barriers to Use, which described aspects of the intervention that participants believed could prevent themselves or other young men from participating in the intervention or deriving maximum benefit from it. What follows are some of the most frequently endorsed or most salient of these critiques. Because the intervention was updated between waves of participants, the number of participants endorsing any particular suggestion is not meaningful as a fraction of the sample size, and is thus not reported.

#### Functionality

Some participants indicated it was unclear from the home screen which features of the app they were supposed to use each day, since there was nothing to indicate which Daily Scoops and tools were unused or newly available. In response, the app was updated to add a glowing border around new or unaccessed material, visually guiding the user to content of interest. The participants who provided this feedback were among the first half of usability testers. Participants in later waves no longer expressed the same concern. One advantage of performing revisions on a rolling basis during testing was being able to see that users’ concerns were being addressed.

Another concern that youth expressed was that providing a graph displaying mood over time could potentially be upsetting if it showed that their mood had been deteriorating. This is a risk of providing visualizations of users’ mood data. We hope coaching will mitigate the possibility of the mood graph creating the iatrogenic effect of further demoralizing a user whose mood declines despite using the app regularly enough to provide mood ratings. The coach can discern with the youth what might be contributing to his mood decline and which features of the app (eg, an In-the-Moment tool) might help the youth to cope better. If concerns remain once the app has been evaluated in the field, we will consider removing the graph or making it accessible only to the coach, who could apply clinical judgment in choosing whether to share it with a given user.

#### Personalization

In general, participants did not react positively when they were asked to enter personal data into the app, and the app did not in return provide some kind of tailored response. One participant said the lack of personalized feedback made the Social Support tool (designed to help a youth assess his levels of various types of social support) feel like “just some survey” and that he thought it would be used more if it gave some kind of feedback based on the values entered. In response, we updated the Social Support tool to provide 24 unique combinations of feedback based on the unique needs of the user himself, suggesting how he might broaden or deepen his social support.

Another aspect of the intervention that participants found to be impersonal was that the asking of some questions seemed to not make sense based on information already entered by the user. An early version of the Mood Rater asked the user to endorse how intensely he was experiencing several emotions (eg, “How sad are you right now?”). One tester commented that if they had already endorsed sadness, it felt like they had not been heard when they were also asked, “How happy are you right now?” With the understanding that one can be happy and sad at the same time, we did agree that the sequence of questions was needlessly complex. We addressed the concern by streamlining the Mood Rater and replacing those items with two questions: one that asks the user to rate his mood valence on a scale of “very bad” to “very good,” and one which presents a list of emotions and asks the user to check off those he is experiencing. This removed a source of impersonal-feeling content and reduced the amount of time necessary to complete the Mood Rater.

**Figure 4:**
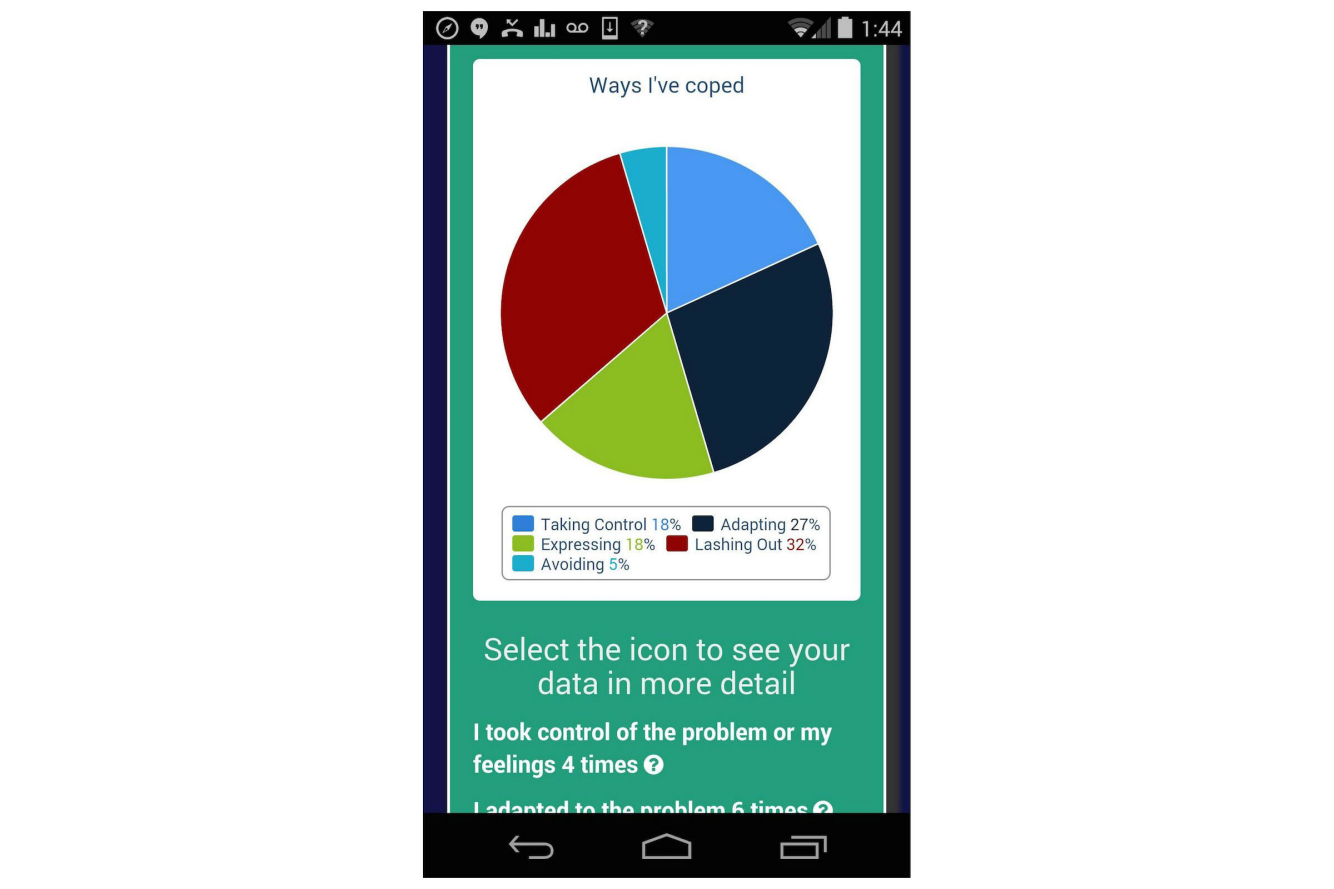
Categories of coping strategies endorsed in the Daily Review.

At the same time, some youth wanted to be able to provide a fuller account of how they were feeling, and why, than the Mood Rater allowed. While we were streamlining the Mood Rater, we also created the Daily Review, intended to be used once each evening, to give the user a chance to reflect on high and low points of their day, what triggered them, and how they responded to those situations. The addition of the Daily Review also addressed the concern inherent to ecological momentary assessment (EMA) [44] that periodic assessment of current states may fail to capture some important events. Figure 4 shows a visualization displaying the types of coping strategies the user tends to use, gathered during the Daily Review.

#### Presentation

Feedback on the methods used to deliver information tended to be more idiosyncratic, with each tester expressing personal preferences regarding how they would prefer to engage with the app. Even so, some themes recurred in feedback from the youth which did guide further development of the intervention in this area.

One common theme was that the Daily Scoop material was perceived as being too lengthy, despite our attempts to keep it brief from the outset. Aside from perceptions that there was too much written material, one participant stated straightforwardly that he would “never” read it because doing so felt like homework. Some users expressed a preference for the inclusion of audio or video to replace or supplement the written Daily Scoops. In response, we did supplement some Daily Scoops with audio and video. We added spoken versions of some of the Daily Scoop content, making some Daily Scoop content accessible by listening rather than reading. We also added short videos to some of the first Daily Scoops, where it seemed especially important for users to grasp the concepts presented in order to derive maximum benefit from later material. The videos depicted a male in his late twenties talking about his own personal experiences that related to the concepts being presented. Testers had mostly positive responses to these videos. Some feedback indicated a sensitivity to the notion that video content was scripted or inauthentic in some way. However, this did not seem to render the videos useless, as one youth with authenticity concerns regarding one video also reported a positive reaction to its message.

Early usability testers were exposed to test videos produced using different paradigms (animated characters discussing Daily Scoop concepts, a solo actor acting as a guide to the concepts, and vignettes with several actors demonstrating the concepts) to assess general format preferences. Participants expressed a diversity of opinions, but there were some commonly repeated themes. Several youth thought the single actor videos were more effective. Usability testers in subsequent waves were shown more single-actor videos, which continued to be better received than video vignettes with multiple actors. Some participants commented on how attractive the characters in the video were:

He was cute, so that was a plus for keeping me interested in the video.

Participant #4, 19-year-old gay Latino male; high-school equivalent education

Attractive characters may not be enough to maintain engagement, however. One participant who found one of the animated characters to be “gorgeous” still said he found the video boring.

It seems that presenting key material as concisely as possible should be a guiding principle when developing material for this population. It also became evident that simply replacing text with multimedia content would not have solved this problem. Some participants stated a preference for reading the didactic material, one stating that in general he is only interested in watching videos when a visual is required for learning. Some participants suggested that the Daily Scoops be presented as text supplemented with other media:

I kind of grew up watching like the “It gets better” videos...my generation maybe relates more to watching videos and stuff. But I think that you could honestly do both. I think like have a video link and have the story. There are so many times that I can't watch a video and I'll just want to read it, so I think that having both would be cool.

Participant #2, 19-year-old gay white male; college student

Since preference for media type varied from individual to individual, it appeared that presenting information in more than one medium would maximize a given user’s likelihood of engaging with it. Whereas it may not be appropriate or feasible to provide all intervention content in multiple modalities, the feedback we received indicated that adding multimedia content when feasible would maximize the impact of didactic material.

#### Aesthetics

Feedback on the app’s aesthetics played a crucial role in helping us determine what would be the most broadly appealing to this population. A substantial number of our earlier usability testers stated that the colors should be brighter. We made the app’s background solid and dark blue so it would provide high contrast to the colors used for buttons, images, and other parts of the user interface, and we replaced colors that were dull or did not stand out from the background. This effort was ultimately rewarded with an overall aesthetic that is more cohesive across the app and appears more lively. After these changes, subsequent usability testers no longer offered any critiques regarding the app’s aesthetics.

Another set of aesthetic issues that came up several times related to the fact that early versions of TODAY! were running as a Web application on a mobile phone browser. Depending on how a user interacted with the screen, sometimes the browser’s URL bar would appear and reveal this fact. This happened in several early usability sessions. One participant said the URL bar made the app “ugly” and urged us to offer the intervention as a “legit app” instead. Another youth (not referencing the URL bar) also expressed a preference for an app that was not running in the browser. It is notable that the youth were not commenting on content at all. In one case, the accidental discovery that the app was running inside the mobile browser provoked the strong reaction, and in the other, it was just the idea that it was running inside the mobile browser that elicited a preference for a different paradigm. These participants did not articulate exactly what felt illegitimate to them about the intervention being delivered as a Web application. TODAY! is now packaged as a standalone Android app like the apps in an Android app store.

#### Lesbian, Gay, Bisexual, and Transgender (LGBT) or Youth Content

Despite taking care from the outset to design TODAY! for young sexual minority men, usability testers still had a wealth of suggestions for topics they would like to see addressed in this kind of app. One of the most often-suggested topics was coming out:

because...you obviously come out to your parents like one time, but you have to come out on like a daily basis as a gay man. It kinda wears on you or you’ll be in a situation like I went to a new school for a year and then I transferred and then I had to like do it all over again and it was like, you kinda forget a little bit...

Participant #2, 19-year-old gay white male; college student

**Figure 5:**
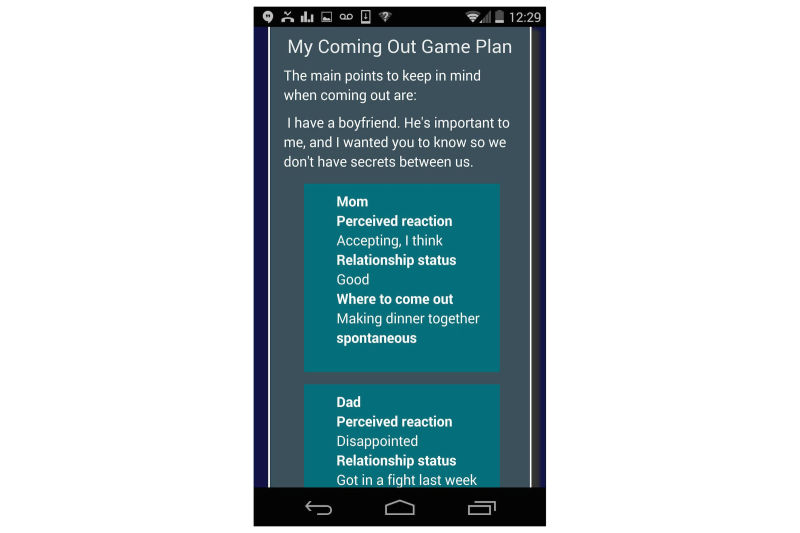
The Coming Out Game Plan tool after a user has entered data.

The young men that TODAY! is intended to help do not necessarily identify as gay or bisexual, and it is possible this material would not be helpful to all users of the app. The relationship between disclosure of sexual orientation and well-being is also somewhat complex [45], and addressing this issue clinically, particularly with this age group, is not simple. We are certainly in no position to recommend any particular course of action to any individual. Thus, we added eight optional Daily Scoops and three tools that deal solely with aspects of coming out, including assessing available social support and weighing all options. For youth who choose to come out, the supplemental material covers preparatory steps, the disclosure itself, and coping with potential consequences. Our goal was to provide a structure whereby a youth could thoughtfully determine for himself whether coming out is the best choice at this time, and if so, prepare in a comprehensive way. Figure 5 shows the Coming Out Game Plan tool being used.

Another frequently mentioned topic was social isolation or inadequate peer social support. These issues are especially salient in a population who may feel set apart from their peers, who may conceal their sexual orientation, and who may have limited contact with other sexual minority youth. In response, we highlighted the Social Support tool that was already part of TODAY!, encouraging youth to return to it and reassess their levels of support as they move through the program. This feedback came while the Social Support tool was already being updated to give personalized feedback (see Personalization, above), so it now can offer ideas about shoring up social support in areas where it is lacking. We also added a new category of In-the-Moment tools to manage loneliness. These tools focus on ways one might reach out to others when feeling lonely, as well as making the most of time alone.

Another feature that youth often mentioned as desirable was a social networking component. One repeated suggestion was that this could be a forum where they could discuss issues and receive tips from peers:

Like they say like I’m feeling this kinda way...like an instant message thingy and we’d be able to talk to them through that...they don’t know who we are, we don’t know who they are...like [some screen name] said this and I feel much better...I’m dealing with the same situation...It’s like a message board..

Participant #5, 19-year-old gay black male; high school graduate

Another idea floated was a messaging system where users of TODAY! could receive support from other users after posting about their day or how they were feeling. One youth stated he would like to share his accomplishments within the app with other app users. Although we lacked the resources to add social networking features at this time, we intend to provide an indirect connection between users by allowing participants to volunteer a narrative about their experience with TODAY! each week. Narratives perceived by study staff to be of potential value to other users will be deidentified and made visible to other users.

#### Barriers to Use

The usability testers were also queried about factors that might hypothetically prevent themselves or other young sexual minority men from using and staying engaged with the TODAY! program. The topic that came up most frequently as a possible barrier was the idea of having weekly phone calls with the coach. A number of our participants indicated scheduling this call could be difficult due to their schedule, the desire to remain discreet, or other factors:

Me personally, I probably wouldn’t want to use the coach because if it’s an app...like everything will be on the phone. Cause if I’m at work or something and I want to use it, I may not have time to talk to someone...maybe that could be an optional thing in the app...I don’t think it should be mandatory cause some people...may be benefiting by just using the app. Or maybe they don’t feel comfortable talking to someone.

Participant #1, 20-year-old gay black male; has trade school certificate

In response, we have made it possible for the youth to contact their coach via telephone, text message (SMS), or email to communicate between calls or schedule their phone conversations, which can take place at different times each week according to the user’s schedule. Relating to the coach, being able to open up with the coach, and possibly not getting along with the coach were also concerns. A participant who was concerned about the relatability of the coach said that it might help just to see the coach’s face. We created a video of the coach introducing herself to the participants and integrated it into a “Meet the Coach” section of the intervention. We hope that revealing the person behind the word “coach” early in the program will help youth feel more comfortable with the idea. Although several participants voiced concern about some aspect of the coaching protocol, some offered suggestions to mitigate those concerns.

There are a few other possible barriers worth noting. As mentioned above (see Presentation), one usability tester stated outright that he would not look at any of the Daily Scoops because they felt like homework. Another youth believed that using certain tools (eg, the Thought Record and Problem Solver) could get tedious, so he did not think he would use them. Understanding that individual differences may lead different participants to prefer Daily Scoops to using tools or vice versa, we have since tried to make the tools as usable as possible on their own, adding introduction screens to each tool with brief summaries of the tool’s purpose and the concepts it employs. Although we expect individuals who make use of both the didactic material and the tools to remain the most engaged and have the most success, we also want individuals with different preferences to derive as much benefit from the app as possible.

#### Positive Feedback

Whereas all participants had positive reactions to the app, individual participants believed different aspects of TODAY! would be the most helpful to them, personally. The graphs providing feedback based on Mood Rater and Daily Review input were cited by one user as the most helpful feature; he believed seeing the patterns in his mood could over time help him learn how to improve his mood in the future (but also see above, Functionality, for concerns about the mood over time graph). One participant was most positive about the Daily Scoops, which he found to be informative. He also found great value in the community resources referenced in the app:

I didn’t even know there was a hotline for lesbian, gays, bisexuals. It’s very informative.

Participant #5, 19-year-old gay black male; high school graduate

Another commented on the Lifesaver button and said it was a great idea. Others cited the tools as most likely to be helpful. One agreed that the Toolbox sounded useful but believed that he himself would only respond well to the In-the-Moment tools.

That each individual highlighted different aspects of TODAY! as being the most helpful validates to some extent the comprehensive design of the intervention. One participant commented on this directly:

I don't think I’ve seen such a comprehensive stress, anger, depression app like this. It’s a really great idea.

Participant #3, 19-year-old gay white male; college student

## Discussion

### Principal Findings

The participants expressed enthusiasm for a comprehensive mobile phone app designed to treat clinically significant symptoms of anxiety and depression among young sexual minority men. These young men tended to prefer bright colors, presentation of didactic content in multiple media formats, brief text content, personalized feedback, and features allowing them to record their mood quickly, yet with options to provide deeper responses. Some of them further suggested that caution should be taken when presenting visualizations of mood data due to the potential to demoralize youth whose mood is negative or deteriorating. Participants also indicated that it was very important to them that an intervention culturally tailored for young sexual minority men like themselves address the topics of social isolation and coming out.

It also became clear that an intervention that intends to include human supports meant to increase engagement (such as a coach) might overcome many obstacles to participation by introducing those supports early on and facilitating flexible scheduling with them. Participant feedback also suggested that future studies should explore the potential for social networking features to enhance such apps. Providing a peer network would pose many potential challenges that would have to be considered [46]. However, given the frequently voiced concern about isolation and the importance of social support, having such a feature may be of benefit.

### Human Supports

The feedback we received on human supports raises a bigger issue that reaches beyond the specifics of our intervention and targeted population. Our usability testers raised many potential problems they saw with having an expert coach guide them through the intervention, and at the same time requested that they be able to interact in some way with peers who are using the same intervention. Adding a social networking feature was beyond the scope and budget of this version of TODAY!, but we intend to explore the possibility of adding social networking to future versions; we have begun preliminary research to attempt to determine the broad outlines of a social networking feature that would be appropriate, effective, and meet youth’s expectations of peer interaction. Meanwhile, requiring coach support as we currently do does potentially restrict access to the intervention relative to a standalone mobile phone app with no clinician involvement. If expanding access to care is one of our goals, and feedback from usability testers has been mixed, why do we not consider removing or replacing the coaching protocol? We know from the broader literature that eHealth interventions for anxiety and depression have been plagued by low levels of adherence that limit their potential [47] and that addition of human coaching protocols increases adherence [48] and efficacy [49]​ in the general population. There is unfortunately no formula to guide us in determining the appropriate balance between what the literature to date tells us, what individuals from a target population tell us about themselves, and our goal to expand access to those who are presently underserved. This decision thus highlights a fundamental tension that can arise in usability testing.

### Generalizability

The other known technology-delivered CBT intervention for young sexual minority individuals with depressive symptoms, Rainbow SPARX, targets depressive symptoms only, is presented as a computer game, and was tested in non–gender-specific sexual minority youth in New Zealand. In contrast, the TODAY! app targets symptoms of depression and anxiety, is presented as a mobile phone app, and was tested in sexual minority males in the United States. Even so, most of the usability feedback received about Rainbow SPARX that differed substantially from feedback we received on TODAY! was based on these differences in intervention format and target population, or on items very specific to one intervention or the other. In any domain they could both be evaluated in, the youth who usability tested TODAY! and the youth who participated in focus groups and feasibility trials for Rainbow SPARX gave similar feedback on these two different interventions [17,50]. Areas where similar feedback were given include that both were generally received positively, youth requested that more be included in both about coming out, and testers for both emphasized in some way the value of friendships and community in coping with their problems [50]. The similarity in feedback regarding quite different interventions lends support to both sets of findings and indicates they may be generalizable to a broader population of sexual minority youth.

### Consistency With Recently Published Guidelines

The positive reception given TODAY! by the young men in our study supports the value of tailoring interventions specifically to young men who are attracted to men. This inclusiveness was always a guiding principle in the design of TODAY!, but two of the recently published recommendations for tailoring eHealth interventions for sexual minority individuals [30] that did not guide the development of this intervention did arise during usability testing, lending support to their importance. First, while we do stress the importance of social support and have an entire tool devoted to assessing and enhancing social support, we did not originally consider a social component to the intervention itself. A social networking component was recommended by several of our participants during usability testing. Second, while the entire intervention is tailored for young men who are attracted to men, we did not originally intend to include significant material devoted to the topic of coming out. Early feedback we received from participants made it clear that this information was highly desired.

### Limitations

Eligibility requirements for our usability testing sample required the presence of only mild depressive or anxious symptoms on a brief screener, whereas the intervention is intended to treat symptoms that are clinically significant. The more mild nature of the symptoms endorsed by many youth in the current sample was reflected in one case by a usability tester declaring he would not be likely to use TODAY! simply because he was “not that depressed.” This should be taken into consideration when interpreting the responses in this study, especially when generalizing them to youth with more severe symptoms of depression or anxiety.

Another limitation of the study is that we reached saturation after only 9 youth had participated. Small sample sizes may be effective in uncovering most usability issues [51,52] when the total number of testers is distributed into waves of 3-5 as in this study. More waves, as opposed to more testers per wave, are of greater benefit due to features of the iteration process [52]. Similarly, a review of usability testing studies using the think-aloud protocol indicated that nine testers can detect approximately 80% of usability problems [53]. However, these conclusions have been criticized [54], and thus, the small sample size may have resulted in failure to detect important usability issues. Other, more definitive sampling issues are that none of the youth in this study identified as bisexual, the sample was divided between black and white participants but did not include multiple Latino youth or any youth from other racial backgrounds, and our results also may not generalize to sexual minority youth living in other cities or in nonurban areas.

Finally, the feedback that we elicited reflects the participants’ personal preferences. There may be a disparity between stated user preferences during our usability sessions and actual usage behaviors and mental health outcomes.

### Conclusions

TODAY! is a comprehensive, culturally tailored mobile phone app for symptoms of anxiety and depression and constitutes one of the first steps forward in the call for technology-delivered, transdiagnostic minority stress treatment [12], and it elicited positive responses from young sexual minority men. Their critiques of the app may also prove useful in the development of other apps for this population. Such apps may be more helpful and appealing to young sexual minority men if they feature bright colors, personalized feedback, brief content, and the options to obtain content through multiple media, as well as to provide depth in their responses to queries. Developers of behavioral apps for young sexual minority males should also consider addressing social difficulties and challenges related to coming out. Finally, future studies should explore the potential for social networking features to enhance such apps.

## Appendix

Multimedia Appendix 1 The interview guide used to conduct the semistructured interviews that were the source of the data analyzed.

## Abbreviations

CBT: cognitive behavioral therapy
COREQ: consolidated criteria for reporting qualitative research
EMA: ecological momentary assessment
GAD-7: Generalized Anxiety Disorder 7-item scale
HIV: human immunodeficiency virus
IRB: institutional review board
LGBT: lesbian, gay, bisexual, and transgender
PHQ-4: Patient Health Questionnaire for Depression and Anxiety
PHQ-9: Patient Health Questionnaire for Depression 9-item scale
SD: standard deviation
SMS: short message service
