# Supplementary material for: Usability of a Culturally Informed mHealth Intervention for Symptoms of Anxiety and Depression: Feedback From Young Sexual Minority Men
Source: JMIR Hum Factors. 2017 Aug 25;4(3):e22. doi: 10.2196/humanfactors.7392 (PMC5591407; doi:10.2196/humanfactors.7392)
Supplement: Multimedia Appendix 1 [file humanfactors_v4i3e22_app1.pdf]

ID # ____________
Date:____________
Time:____________

Usability Testing: Youth
Interview Guide

1.) To start off, I'd like to tell you about the program we're designing and get your initial reactions to it. [DESCRIBE INTERVENTION]
    a. What do you think about a program like that?
    b. What would be good about that kind of program?
    c. What would be bad or difficult about that kind of program?
    d. What kinds of topics do you think would be important to include in a program like that?

2.) We are thinking about having a coach help people through this program. Here's how it would work. First, this coach would have a discussion with the person for about 30-45 minutes over the phone, to get to know them and understand the issues they'd like to work on while they're in the program. Then, every week, the coach would talk with the person for 5-10 minutes over the phone, to encourage them, keep them motivated, and help them with anything that's difficult or confusing about the program. The coach would also email people once a week to check in, and people in the program could email their coach more often if they had questions or wanted extra support.
    a. What do you think about that kind of coaching?
    b. What would be good about that kind of coaching?
    c. What would be bad or difficult about that kind of coaching?
    d. Is there anything about the coaching that you think should be different?
    e. Would you want the phone calls to be longer or shorter?

3.) If you wanted help for anxiety or depression, would you consider taking part in a program like that?
    a. [If NO] What are some reasons you would not be willing to participate?
    b. [If YES] What are some challenges you'd face? What would make it easier to stick with it?

4.) Now I'd like to show you [paper prototypes / a working version] of the mobile phone application and website. While you review these, I'd like you to "think aloud," or say what you're thinking, as you go. That includes all your thoughts about the program, like, "I don't agree with this part," or, "I really like the way this screen looks." It also includes any questions you run into, like "Hmm, where would I go from this screen," or "I'm not sure what this means." By thinking out loud, we can understand your reactions to the material, determine whether anything we've written was confusing, and learn about any parts are difficult to use. Any questions about that?

    It's important to know that we're testing the program, not you. We're trying to make a program that is easy to use. So when any parts are confusing, it just means that the

program is a “work in progress” and we need to improve it. Any positive or negative feedback you can tell us will be extremely helpful, and we appreciate it.

[AFTER THE THINK ALOUD PROCEDURE IS FINISHED]

Now I’d like to ask you some questions about the program materials you just reviewed.

- What part of the program was most helpful? Why?
- What part of the program was least helpful? Why?
    - [IF UNCLEAR] Is there any part of the program that you would never use?
    - [IF UNCLEAR] Is there any part of the program that you would never use?
- What parts of the Daily Scoops were confusing?
- Are there Daily Scoops on other issues or topics that we should consider adding to the program?
- What parts of the tools were confusing?
- Do you have ideas for other tools that we should consider adding to the website?
- Is there anything that could be improved, that would make youth want to come back to use the program more often?
- If you were in a program like this, how many times a day do you think you would be willing to rate your mood on the mobile phone?
- Is there anything else that the program should do?
- Are there any other web sites or phone applications you’ve seen that give the same type of information?
    - How did they compare to this program?
- Do you think that if this program was 10 weeks long, with a new topic and tool each week, would that be too short, too long, or just right? Why do you think that?

General open ended questions:

- **What did you like and dislike about the:**
  - Navigation, or the way you could move around and find things
  - Way the program looked, and its colors
  - Daily Scoops
  - Tools
  - In the moment tools
  - Mood Rater
  - Graphs
  - Inspiration page
  - Community Resources page
  - Life Saver page

Is there anything we haven’t talked about that is missing from this program, that you think should be added?

Is there anything else you think we should know so we can make this program better?

Wrap-up

In closing our discussion today, I’d like to check in with you, because it can be uncomfortable to talk about anxiety and depression. How are you feeling?

Was there anything that bothered you about what we talked about today?

[IF THE PARTICIPANT REPORTS ANY DISCOMFORT, MAKE SURE THEY’RE NOT HAVING ANY THOUGHTS ABOUT HARMING THEMSELVES. OFFER TO TALK WITH THEM ABOUT THEIR CONCERNS AND SEE IF THERE ARE SPECIFIC RESOURCES THAT MIGHT HELP. SEE IF THEY CAN PLAN TO DO SOMETHING RELAXING AFTER THE SESSION, SUCH AS TAKE A WALK OR GET TOGETHER WITH A FRIEND.]

[THANK THE PARTICIPANT AND GIVE THE PARTICIPANT THE RESOURCE LIST, THEIR COMPENSATION, AND THEIR COPY OF THE CONSENT FORM. REITERATE THAT THEY CAN CONTACT US IF THEY HAVE ANY QUESTIONS OR CONCERNS AFTER THE STUDY.]
